# Pitfalls in mutational testing and reporting of common *KIT *and *PDGFRA *mutations in gastrointestinal stromal tumors

**DOI:** 10.1186/1471-2350-11-106

**Published:** 2010-07-04

**Authors:** Sabine Merkelbach-Bruse, Wolfgang Dietmaier, Laszlo Füzesi, Andreas Gaumann, Florian Haller, Julia Kitz, Antje Krohn, Gunhild Mechtersheimer, Roland Penzel, Hans-Ulrich Schildhaus, Regine Schneider-Stock, Ronald Simon, Eva Wardelmann

**Affiliations:** 1 Department of Pathology, University of Bonn Medical School, Sigmund-Freud-Str. 25, D-53127 Bonn, Germany; 2 Department of Pathology, University of Regensburg, Franz-Josef-Strauß-Allee 11, D-93053 Regensburg, Germany; 3 Department of Gastroenteropathology, Georg-August University Göttingen, Robert-Koch-Str. 40, D-37075 Göttingen, Germany; 4 Department of Pathology, University Medical Center Hamburg-Eppendorf, Martinistr. 52, D-20246 Hamburg, Germany; 5 Institute of Pathology, University of Heidelberg, Im Neuenheimer Feld 220/221, D-69120 Heidelberg, Germany; 6 Department of Pathology, Otto von Guericke University, Leipziger Str. 44, D-39120 Magdeburg, Germany; 7 Department of Pathology, University of Erlangen, D-91054 Erlangen, Germany; 8 Department of Pathology, Albert-Ludwig University, D-79098 Freiburg, Germany

## Abstract

**Background:**

Mutation analysis of *KIT *and *PDGFRA *genes in gastrointestinal stromal tumors is gaining increasing importance for prognosis of GISTs and for prediction of treatment response. Several groups have identified specific mutational subtypes in *KIT *exon 11 associated with an increased risk of metastatic disease whereas GISTs with *PDGFRA *mutations often behave less aggressive. Furthermore, in advanced GIST disease with proven *KIT *exon 9 mutation the doubled daily dose of 800 mg imatinib increases the progression free survival and is now recommended both in the European and the American Guidelines. In Germany, there are still no general rules how to perform mutational analysis.

**Methods:**

When comparing results from six different molecular laboratories we recognized the need of standardisation. Six German university laboratories with experience in mutation analysis in GISTs joined together to develop recommendations for the mutation analysis of the most common and clinically relevant hot spots, i. e. *KIT *exons 9 and 11 and *PDGFRA *exon 18. We performed a three-phased interlaboratory trial to identify pitfalls in performing molecular analysis in GISTs.

**Results:**

We developed a design for a continuous external laboratory trial. In 2009 this external trial was conducted by 19 laboratories via the initiative for quality assurance in pathology (QuiP) of the German Society of Pathology and the Professional Association of German Pathologists.

**Conclusions:**

By performing a three-phased internal interlaboratory trial and conducting an external trial in Germany we were able to identify potential pitfalls when performing KIT and PDGFRA mutational analysis in gastrointestinal stromal tumors. We developed standard operation procedures which are provided with the manuscript to allow other laboratories to prevent these pitfalls.

## Background

Gastrointestinal stromal tumors (GISTs) represent the most common mesenchymal tumors of the gastrointestinal tract. About 50% of GISTs behave clinically aggressive. Since 2001, treatment options have dramatically improved with the introduction of tyrosine kinase inhibitors. On the molecular level, the vast majority of tumors carries activating mutations in the *KIT *gene or the *PDGFRA (platelet derived growth factor receptor alpha) *gene, both genes encoding closely related type III tyrosine kinases. The relevance of the mutational status in these genes both for clinical prognosis and for prediction of response to treatment has been increasingly recognized[1,2]. Mutational analyses are now performed in many laboratories worldwide and several protocols have been published. Given the high impact on clinical decisions, mutational testing should meet the highest standard for quality assurance.

The reported frequencies of mutated sites differ considerably between anatomic locations of tumors and depend on the setting of calculation (e.g., population based study vs. results of clinical trials)[3,4] but also on technical issues. Primary activating mutations occur in the extracellular parts of the receptor protein (i.e., *KIT *exon 9), in the juxtamembrane domain (*KIT *exon 11, *PDGFRA *exon 12), in the first tyrosine kinase domain *(KIT *exon 13, *PDGFRA *exon 14) or in the second tyrosine kinase domain (*KIT *exon 17, *PDGFRA *exon 18). As a strongly simplified rule one can suppose that about 65% of all GISTs harbour primary *KIT *exon 11 mutations, whereas *KIT *exon 9 and *PDGFRA *exon 18 mutations account each for about 10% of primary mutations. Thus, about 85% of all GISTs carry a mutation at one of these three sites. Approximately 10% of GISTs are so-called wildtype GISTs without any detectable mutations in the known hot spots. The remaining 5% may carry mutations in the exons 13 or 17 of *KIT *or in exons 12 or 14 of *PDGFRA*, so the frequency in each of these regions is below 1% [5,6]. For these latter regions there is no experience concerning their prognostic and predictive value, therefore we decided to restrict our study to the most important and clinically relevant exons, i. e. *KIT *exons 9 and 11 and *PDGFRA *exon 18.

Since fresh frozen tumor tissue is only rarely available for mutational testing, formalin-fixed and paraffin-embedded tissue (FFPE) is widely used for molecular analyses. The integrity and stability of DNA in FFPE is the limiting factor for the reliability of mutational testing [7]. DNA quality depends predominantly on the manner and duration of fixation and the age of paraffin blocks.

Our results have been generated by Sanger sequencing as in our view, it remains to date the gold standard for mutational analysis of *KIT *and *PDGFRA *and is already available in nearly all places. Denaturing high-pressure liquid chromatography (DHPLC) as used successfully by some other laboratories[2] is a screening technique which has to be followed by direct sequencing as it cannot predict the precise sequence of the highly variable *KIT *and *PDGFRA *mutations occurring in GISTs. DHPLC is not available in all places due to high costs for the technical equipment. Next generation sequencing systems that work with pyrosequencing techniques or mass-spectroscopy are at the moment not suited for routine mutation analysis because they are designed for high-throughput sequencing, still too expensive and not widely available.

We aimed to analyze all steps of mutational testing, beginning with DNA extraction from FFPE samples up to the correct reporting of mutations to develop standard operation procedures suitable for every laboratory willing to establish sequence analysis. Therefore we compared the analysis methods of six German university laboratories. In a first step, the participating laboratories examined the reliability and comparability of *KIT *exon 9 and 11 and *PDGFRA *exon 18 mutation testing using a set of 10 DNA samples extracted from FFPE tissue. Second, to consider the heterogeneity of tissue blocks from different sources, a set of 12 tissue blocks collected from the participating laboratories was analyzed. The third step served for the detection of the ideal primer combinations for mutation analysis in the different hot spots. In all steps of the internal trial, each of the participating laboratories used its own protocols. By standardizing the methods as a result of the different trial steps, a high degree of interlaboratory concordance could be achieved.

Besides the results of our trial, we here present a cross-validated protocol for testing and reporting the most common mutations in GISTs. Additionally, the results of an external trial for other laboratories are presented underlining the need of our initiative.

## Results

### First trial (mutation analysis from extracted DNA)

The results for the mutation analysis as provided by the panel labs are shown in detail in Table 1. All results were returned within the time limit. The sequence data obtained by Lab A at a previous and the actual analysis were supposed to be the nominal results; in case of variance compared to the other panel labs the data were reanalyzed and adjusted (as for sample T1.4).

**Table 1:** Nominal and actual results of the mutation analyses of *KIT *exons 9 and 11 and *PDGFRA *exon 18 performed in trial 1.

sample	exon	nominal result	Lab A	Lab B^a^	Lab C^a^	Lab D	Lab E^a^	Lab F
T1.1	*KIT *11	c.1670_1675del6						
		p.W557_V559delinsF			p.W557_K558del		p.W557_V559del	

T1.2	*KIT *9	c.1510_1515dup6						
		p.A502_Y503dup						

T1.3	*KIT *11	c.1714_1740dup27						c.wild-type
		p.D572_H580dup						p.wild-type

T1.4	*KIT *11	c.wild-type					c.1690_1692del	
		p.wild-type					p. N564del	
	*PDGFRA *18	c.2527_2538del12			c.wild-type	c.2464G>A	c.wild-type	
		p.I843_D846del	p.D842_H845del		p.wild-type	p.R822H	p.wild-type	

T1.5	*KIT 9,11*	c.wild-type						
	*PDGFRA 18*	p.wild-type						

T1.6	*PDGFRA *18	c.2525A>T						c.wild-type
		p.D842V						p.wild-type

T1.7	*KIT 9,11*	c.wild-type						
	*PDGFRA *18	p.wild-type						

T1.8	*KIT 9,11*	c.wild-type						
	*PDGFRA 18*	p.wild-type						

T1.9	*KIT *9,11	c.wild-type						
	*PDGFRA 18*	p.wild-type						

T1.10	*KIT *11	c.1676T>A					c.wild-type	
		p.V559D					p.wild-type	

Only the nominal results of the relevant exons are given, other exons are wild-type; for the individual panel labs only the actual results differing from the nominal results are denoted on DNA and protein level; matching results are not shown.

^a ^changes in DNA sequence were not specified in Labs B, C and E

**Figure 1:**
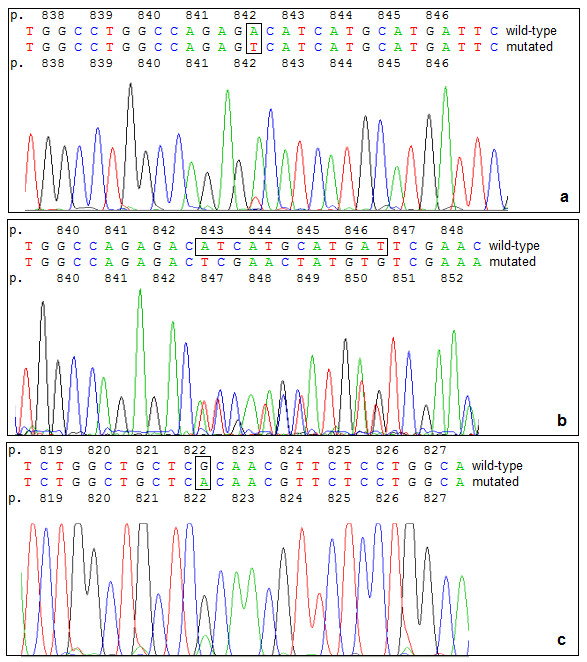
**Sequence details illustrating the divergent results of the first trial**. **a **Detail of the forward sequencing reaction of sample T1.6: the missense mutation c.2525A>T leading to p.D842V in *PDGFRA *was not detected due to high background peaks. **b **Detail of the forward reaction of sample T1.4: the deletion c.2527_2538del12 in *PDGFRA *was misinterpreted as p.D842_H845del instead of p.I843_D846del. **c **Detail of the forward reaction of sample T1.4: Reamplificaton lead to the assumption of an unknown missense mutation in *PDGFRA *(c.2464G>A leading to p.R822H). Mutated nucleotides are marked by open boxes.

For five out of 10 samples (T1.2, T1.5, T1.7, T1.8 and T1.9), all six laboratories achieved identical assessments. In five samples, conflicting data were obtained by at least one of the panel labs. For these samples, the reported data were compared to the corresponding sequence electropherograms first. In one sample (T1.1) the divergent mutation data on *KIT *exon 11 obtained by two labs could be clarified by re-evaluation of the electropherograms. Concerning the samples T1.3, T1.6 and T1.10, there was accordance in five labs, one lab each failed to detect the mutation in *KIT *exon 11 or *PDGFRA *exon 18 due to high background peaks (Fig. 1a). Evaluation of mutation data in sample T1.4 was more complex: three labs achieved identical results concerning *PDGFRA *exon 18, in Lab A the electropherogram was incorrectly evaluated (Fig. 1b), and two labs reported a false mutation. In Lab D, reamplification lead to the pretended change p.R822 H in *PDGFRA *exon 18 (Fig. 1c). Lab E reported a mutation in *KIT *exon 11. This was the only example for the allocation of a mutation to a wild-type exon; all other wild-type sequences were judged correctly.

Taken together, analysis of *KIT *exon 11 failed in three cases, including two cases with the detection of wild-type sequences instead of point mutation and duplication, respectively, and one case with the description of a deletion instead of wild-type. The mutational status of *PDGFRA *exon 18 was described falsely in four cases including the detection of wild-type sequences instead of the point mutation D842V and two deletions. In one case, a point mutation was detected instead of deletion.

The discrepancies in mutation analysis from extracted DNA samples had several reasons: First the interpretation of electropherograms had not been discussed prior to the trial, nor were there rules how to report the results. Second, some labs had difficulties in detection mutations, especially point mutations due to high background in their electropherograms. Third, in one lab reamplification led to a false-positive result in *PDGFRA *exon 18.

### Second trial (mutation analysis from FFPE material)

**Figure 2:**
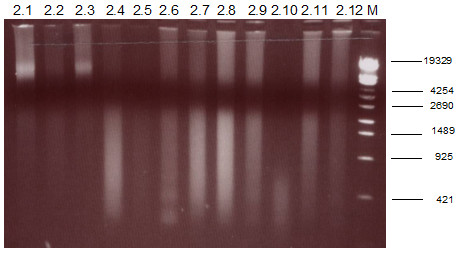
**1% gelelectrophoresis of DNA extracts prepared for the second trial**. M: DNA length standard λ/Eco130I (Fermentas, St. Leon-Roth, Germany).

As detailed in additional files 1, 2, 3 and 4 each of the panel labs followed its own extraction protocol. The quality of DNA extracts was assessed by agarose-gelelectrophoresis which is shown exemplarily for Lab A in Fig. 2. All samples showed the typical DNA-smear with samples T2.5 and T2.10 containing only strongly fragmented DNA and samples T2.2 and T2.4 indicating very low amounts of high-molecular weight DNA. The amount of DNA required for amplification was estimated individually by each panel lab.

The results of the mutation analysis as provided by the panel labs are shown in detail in Table 2. All panel labs met the deadline for reporting the results. Except for sample T2.4 and T2.10 the results of the majority of the panel labs were defined as nominal sequence data. For these two samples the sequence data obtained on frozen tissue by Lab B and Lab E who provided the corresponding tissue blocks were supposed to be the nominal results.

**Table 2:** Nominal and actual results of the mutation analyses of *KIT *exons 9 and 11 and *PDGFRA *exon 18 performed in trial 2.

sample	exon	nominal result	Lab A	Lab B	Lab C^a^	Lab D	Lab E^a^	Lab F
T2.1	11	c.IVS10(-5)_(-1);						
		1648_1672del30					p.Q567_577Pdel	
		p.K550_558del						

T2.2	18	c.2528_2539del12	c.2530_2538del9					
		p.I843T;				p.I843_S847insT	p.I843_S847del	
		M844_S847del						

T2.3	11	c.1669T>A						
		p.W557R						

T2.4	11	c.1712_1738dup27	c.^b^	c.^b^	c.wild-type			c.^b^
		p.H580L;	p.^b^	p.^b^	p.wild-type	p.insLDPTQLPYD	p.D572_H580ins	p.^b^
		D572_H580dup						

T2.5	11	c.1727T>C						
		p.L576P						

T2.6		c.wild-type						
		p.wild-type						

T2.7	11	c.1669T>A					p.W557_R558del	c.wild-type
		p.W557R						p.wild-type

T2.8	11	c.1679T>A					c.wild-type	c.1811T>A
		p.V560D					p.wild-type	p.V604D

T2.9	11	c.^c^	c.1693_1716del24;	c.1693_1720del28;				c.1692_1720del
			1720del	insC	p.G565P;	p.G564_D572del	p.G565_D572del	
		p.^c^	p.G565_D572del;	p.G565_T574delinsP	N566_T574del			p.G565_P573del
			T774del					

T2.10	11	c.1668_1723del57^d^	c.1679T>A	c.1672_1716del45	c.wild-type	c.wild-type		c.wild-type
		p.W557_Q575del^d^	p.V560D	p.K558_D572del	p.wild-type	p.wild-type		p.wild-type

T2.11	11	c.1669_1674del6						
		p.W557_K558del					p.W557_R558del	

T2.12	11	c.1676T>A						
		p.V559D					p.W557_R558del	

Only the nominal results of the relevant exons are given, other exons are wild-type; for the individual panel labs only the results differing from the nominal results are denoted on DNA and protein level, matching results are not shown.

^a ^changes in DNA sequence were not specified in Lab C and E

^b ^in Lab A and Lab F the DNA was not amplifiable; in Lab B mutation was detected from frozen tissue

^c ^complex deletion, different specifications are possible (see Lab A, Lab B and Lab C)

^d ^mutation could not be detected from FFPE material in the second trial; in Lab E mutation was detected from frozen tissue

In three out of 12 samples (T2.3, T2.5 and T2.6), the accurate mutation was found and described correctly by all panel labs. In another three samples (T2.2, T2.9 and T2.11), the accurate mutation was found but reported falsely by at least one lab (sample T2.11) and at most by three labs (sample T2.2). In sample T2.9, only the three different descriptions given by Lab A, Lab B and Lab D were applicable.

In four out of 12 samples a wrong mutation or a wild-type sequence was reported by one (sample T2.1 and T2.12) or two labs (sample T2.7 and T2.8). The allocation of wild-type instead of mutation was most often observed in case of point mutations.

The remaining two samples (T2.4 and T2.10) yielded rather controversial results. The DNA extracted from these samples was either not amplifiable or generated wild-type sequences or even false mutations due to contaminations by other DNA samples. Sequence analysis done on DNA extracted from frozen tissue at a later date was successful in all of the panel labs (data not shown). In the second trial all wild-type sequences were judged correctly.

Taken together, six samples were assessed as wild-type instead of mutation, including two point mutations, three deletions and a duplication. In three samples, a wrong mutation was assigned. This was probably due to contamination or confusion of samples, as all these mutations occurred in other samples of our probe set.

In addition to the results of the first trial, the second trial showed that special attention has to be paid to DNA quality and to samples with an underrepresentation of wild-type or mutated sequences. The possibility of contamination has to be taken into account.

### Third trial (comparison of different primer sets for *KIT *exon 11)

Three different DNA samples containing mutations in *KIT *exon 11 (i.e. two deletions, c.1735_1737delGAT; p.D579del and c.1661_1705del45bp; p.E554_Y568del and one duplication, c.1728_1766dup39bp; p.L576_L588dup) were analyzed. Each of the participating laboratories amplified the DNA from these samples only with four different primer sets for *KIT *exon 11 (Additional file 4, Table AF 2 in the additional files), because the primer pairs used in Lab A and B and Lab C and D were identical, respectively.

The amount of DNA required for amplification was estimated individually by each panel lab. All results were reported within the time limit and are detailed in Table 3. Each lab provided a judgement of amplification and sequencing quality for all primer/sample combinations. All approaches with technically successful performance of amplification and sequencing yielded the expected results.

**Table 3:** Results of the mutation analyses of *KIT *exon 11 performed in trial 3.

	Lab A						Lab C						Lab D						Lab E						Lab F					
Sample	T3.1		T3.2		T3.3		T3.1		T3.2		T3.3		T3.1		T3.2		T3.3		T3.1		T3.2		T3.3		T3.1		T3.2		T3.3	
Experimental Step	A	S	A	S	A	S	A	S	A	S	A	S	A	S	A	S	A	S	A	S	A	S	A	S	A	S	A	S	A	S
Primer Lab A/B	+	+	+	+	+	+	+	+	+	+	+	+	+	+	+	+	+	+	+	+	+	+	+	+/-	+	+	+	+	+	+
Primer Lab C/D	+	+	+	+	+	+	+	+	+	+	+	+	+	+	+	+	+	+	+	+	+	+	+	+/-	+	+	+	+	+	+
Primer Lab E	+/-	-	+/-	-	+/-	-	-	-	-	-	-	-	+	+	+	-	+	+/-	+	+	+	+	+	+/-	+	+/-	+	+/-	+	+/-
Primer Lab F	+/-	-	+	+	+	+/-	+	+	+	+	+	+	+	-	+	+	+	-	+	-	+	+	+	-	+	-	+	+	+	+

For each lab the result of amplification (A) and sequence analysis (S) is detailed. "+" means success of amplification or nominal result in sequence analysis, "+/-" means only faint amplification with unspecific extra-bands or different/not-scorable sequence-analysis, and "-" means failure of amplification or sequence analysis. Primer pairs used in Lab A and B and Lab C and D were the same, respectively. Lab B did not participate in the third trial suppl.

**Figure 3:**
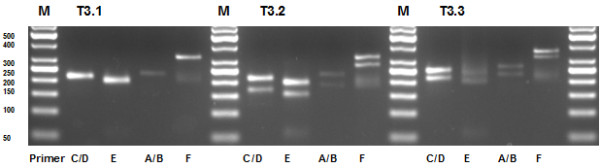
**3% gelelectrophoresis of the PCR products amplified from the third-trial samples**. Amplifications were performed with four different primer pairs in Lab D. The amplification was done using an annealing temperature of 60°C for each primer set. M: DNA length standard Gene Ruler 50 bp (Fermentas, St. Leon-Roth, Germany).

Exemplarily, the amplification products generated in Lab D are shown in Fig. 3. Clear bands for all three samples were observed only in amplification reactions using primer combinations A/B-11 and C/D-11 thereby the bands for the former were rather faint. Amplification with primer pair E-11 resulted in synthesis of primer dimers in each reaction. In sample T3.3, which carries a large duplication in *KIT *exon 11, a clearly visible DNA smear with a larger size than the PCR products could be noticed. Primer pair F-11 produced unspecific fragments sized between 200 and 250 base pairs. With 294 bp, this primer pair amplifies the largest fragment of *KIT *exon 11. Taking together all approaches to amplify the DNA samples with each primer/sample combination in the five panel labs the amplification with primer pair E-11 failed in three instances. Unspecific bands were observed in several PCR products generated with primer pair F-11 but the quality was always sufficient to continue with the next experimental step.

The amplification results were reflected by the sequencing results: in each panel lab all sequencing reactions carried out with primer combinations A/B-11 and C/D-11 were successful. Altogether the sequencing reaction of PCR products generated with primer pair E-11 or primer pair F-11 ended without result in seven or six cases, respectively. This was mainly due to the location of these two primer pairs. The forward primer used in Lab F is too far upstream of the exon-intron boundary resulting in faint fluorescence signals in the end of the analyzed fragment and is therefore not suitable for the detection of distal mutations. Unlike primer pair F-11, primer pair E-11 generates the smallest *KIT *exon 11 fragment in this trial, where the forward primer is located right in the beginning of *KIT *exon 11 and therefore not suitable for detecting mutations in this region (data not shown).

## Discussion

The detection of common *KIT *and *PDGFRA *mutations in GIST is implicated in diagnosis, prognosis, therapy decisions and prediction of response to treatment. Several studies have shown that GISTs are characterized by a strong genotype-phenotype correlation[4]. Among many different types of the heterogenous *KIT *exon 11 mutations, for example, p.W557_K558del indicates a more aggressive clinical behaviour[8,9]. On the other hand, substitutions and duplications at the 3'-end of *KIT *exon 11 are considered to be associated with a clinically less aggressive phenotype[10,11]. In *KIT *exon 9 almost exclusively a single type of mutation occurs, i.e., p.A502_Y503dup, and about 90% of *KIT *exon 9 mutated tumors are located in the small bowel and have a spindled phenotype. These tumors have initially been considered to be at high risk for a malignant course[10,11]. Recent studies have put this conclusion into question[4]. On the other hand, GISTs with *PDGFRA *exon 18 mutations are predominantly gastric tumors with an epithelioid morphology. Among the heterogeneous types of mutations, the point mutation p.D842V is by far the most common. This special type as well as other point mutations and several in frame-deletions are associated with a favourable prognosis[12-14].

The best response to imatinib (standard dose: 400 mg daily) is achieved by GISTs with *KIT*-exon 11 mutations regardless of the type of genomic alteration[1,2]. A very important purpose of mutational analysis of GISTs for treatment options is the detection of *KIT *exon 9 mutants, as several studies have shown that the response rates are significantly improved by applying a higher imatinib dose (800 mg daily)[1,2]. Furthermore, p.D842V, the most common *PDGFRA *mutation, is associated with a primary resistance to imatinib due to structural alterations of the imatinib binding pocket[15]. However, other types of *PDGFRA *exon 18 mutations may respond well to imatinib[6].

For rare cases of negative or ambiguous CD117 (KIT) immunostaining, sequencing of *KIT *and *PDGFRA *can be used for differential diagnosis[16]. The major purpose of mutational analysis is, however, prognostication of the clinical course and prediction of the response to treatment with imatinib, which represents the first line therapy in metastastic and advanced GIST disease[1,2]. In inoperable primary lesions, neoadjuvant treatment may be an option to achieve secondary operability[17]. Very recent studies show the efficacy of an adjuvant treatment in completely R0-resected GISTs to increase progression-free survival[18]. Especially in the adjuvant setting, it may become increasingly important to exclude patients expected to have a mutation leading to a primary resistance to imatinib. Therefore, it is indispensable to establish standardized methodological approaches for sample preparation and sequence analysis as well as for reporting and interpretation of data.

The interlaboratory three-step trial presented here shows on the one hand a high accordance in mutation testing between the six panel labs but on the other hand sources of error occurring in different methodological steps. It is very important to recognize these possible sources of mistakes because of the pivotal role of mutational testing for prognosis and treatment of the individual patient. Summarizing the results of our different trial steps several levels of possible pitfalls can be identified - the practical ones concerning technical procedures and the theoretical ones relevant for data interpretation:

The quality of DNA extracted from FFPE tissue blocks is affected mainly by degradation of target DNA due to the reaction of the phosphodiester backbone with formalin and by the copurification of inhibitory substances as for instance hemoglobin[19]. Some of the parameters which influence the degradation of DNA at the stage of tissue fixation are well known, e.g. the age of specimens, the fixative and the duration of fixation[20,21]. In practice however, these parameters are highly variable, particularly if the material is received from different institutes. The differing results from two samples in our second trial (T2.4 and T2.10) underline the importance of adequate fixation. Our suggestion for optimal fixation is (as published also by others[22,23]) to use 4% buffered formalin for biopsies at least 6 hours, for larger specimens overnight. One major goal of the extraction protocol must be to preserve the DNA as good as possible with a gentle procedure. The different extraction methods the six labs used all proved to be suitable (see Additional files 1, 2 and 4, Methods AF 1.1., AF 2.3. and Table AF 1).

Excess target DNA within a PCR mixture can inhibit the reaction and lead to false negative results due to non-productive target-target interaction[24]. Therefore it is important to assess the DNA amount and the extent of degradation for each sample. For this purpose we highly recommend to use agarose gelelectrophoresis. Unlike the spectrophotometric measurement it allows also the assessment of DNA integrity. Because the PCR result depends on the number of amplifiable fragments it should be avoided to use standard DNA amounts as PCR template. It is strongly recommended not to analyze samples with bad DNA quality. In such cases, additional material (e.g. fresh frozen tissue, if available, or another paraffin block) should be used (see also Additional file 1, Methods AF 1.2.).

Next, the steps of amplification and cycle sequencing implicate some obstacles in generating the correct mutation data. The number of PCR cycles should not exceed 40 as increasing the cycle number can lead to a compensation of poor quality DNA but also increases the risk of amplifiying contamination DNA fragments [25]. It was also shown that reamplification should be avoided even in case of low amounts of PCR products. Reamplification can lead to PCR artifacts and therewith to false allocation of point mutations. One possibility to increase the sensitivity may be the application of nested PCR. However, this additional step prolongs the work-up of tumor samples. If amplification failed twice, the analysis should be stopped at this point. The analysis can be re-tried with another paraffin block (see also Additional files 1 and 2, Methods AF 1.3., AF 2.4.).

The design of the primers in the different genomic regions is another important issue. Too large fragments lead to problems especially in formalin-fixed material. The optimal distance of the forward primer to the exon-intron boundary should be considered in designing the primers. Otherwise mutations in the 3' region or the distal region can hardly be detected either due to high background in the beginning of electropherograms or faint fluorescence signals in the end of the reaction. From the results of our third trial, we decided to recommend two primer pairs for amplification of *KIT *exon 11 which are shown in Additional file 4, Table AF 2.

For the other most frequently mutated exons, i. e. *PDGFRA *exon 18 and KIT exon 9, several observations of the different labs have to be considered before choosing the primer design. In sequencing *PDGFRA *exon 18, it has to be taken into account, that in 11,5% of samples (data not shown) an intron polymorphism (c.2440 - 49delA) can be found. The forward primer can either bind in front of or behind this polymorphism. Whereas in the former case sequence interpretation may be difficult due to interference of sequences, in the latter case the forward primer binds very close to the exon-intron-boundary. Depending on the experience in evaluating sequence data each laboratory has to decide independently which primer design is chosen.

For *KIT *Exon 9 the most frequent mutation p.A502_Y503dup can be evaluated specifically by a shorter 146-bp fragment (as done for example by Lab A referred to as primer pair A-9) from the distal region[10]. By this procedure also the mutation p.F506_F508dup and the complex deletion p.E490_F504del found only once can be detected[26,27]. Alternatively, a primer design including the whole exon 9 can be used detecting also rarely occurring point mutations, e.g. p.P456 S and eventually p.F469L, p.N486 D and p.V489A, which were to date only detected in Ewing sarcoma[28,29]. It has to be kept in mind, that this primer pair generates a fragment of 310 bp in length. Depending on the degree of DNA fragmentation, it may be difficult to amplify this fragment.

A standard protocol for amplification with the recommended primer pairs in all three exons is provided as additional files to the manuscript (see also under additional files 2 and 4, AF 2.4. and Table AF 2).

Interpretation of electropherograms should be done carefully. It is indispensable to analyze not only the base-sequence given by the sequence analysis programs but also to check the electropherograms as many point mutations are underrepresented and thus are easily missed. All wild-type sequences evaluated by eye should be re-analyzed by using an alignment program like "BLAST" (basic local alignment search tool; http://blast.ncbi.nlm.nih.gov/Blast.cgi) to detect homozygous mutations which are easily overlooked in the electropherograms. Mutations should be described according to the standard of the human genome variation society (HGVS)[30]. A comprehensive catalogue of published *KIT *and *PDGFRA *mutation data are available at http://www.sanger.ac.uk/perl/genetics/CGP/cosmic?action=gene&ln=KIT and http://www.sanger.ac.uk/perl/genetics/CGP/cosmic?action=gene;ln=PDGFRA (see also in the additional files 2, 3 and 4, AF 2.5., 3 and Table AF3).

As the results of mutational analyses are the basis of comprehensive clinical decisions, suggestions for reporting the results of mutation analysis are given in Table 4. Thus, these results need to be reported by an experienced pathologist in the context of clinical and histopathological findings. Pathologists and clinicians should ensure that the reports are properly transferred to the responsible physicians. The report should at least include a statement on the individual risk of a given patient and should assess the molecular finding for the therapeutic options.

**Table 4:** Proposal for reporting of mutational results in GISTs

Information required	Optional information
- confirmation of the diagnosis GIST, based on morphological and immunohistochemical findings	

- indicate the type of analysed material (primary tumor, metastasis, local relapse)	- indicate date of surgery if appropriate

- in case of a primary GIST indicate the individual risk classification according to consensus classification [27]	- according to [5]

- report on molecular findings for every exon analysed; indicate mutations on DNA and protein level in a standardized description according to [30]	- indicate homo-/hemizygous mutations - indicate type of examination method (e.g. PCR and DNA sequencing)

- report on expected response to imatinib treatment based on the individual mutation type, according to recent recommendations; - *KIT *exon 9: better response to 800 mg daily - *KIT *exon 11: best response (at 400 mg daily) - *PDGFRA *exon 18: according to special type of mutation (D842V resistant, deletions mostly responsive)	- report on prognostic relevance of the individual mutation type for clinical behaviour, according to recent data, e.g. [28]

- give an individual suggestion for adjuvant therapy, based on the individual mutation type and according to recent consensus recommendations	

The results in our internal interlaboratory trials underline that false-positive and false-negative results in mutation analysis may occur even in laboratories with some experience in this field. The consequences of these mistakes may be dramatical for patients. A false-negative wild type sequence in *KIT *exon 11 in a tumor habouring a mutation in this region could result in a wrong assessment of treatment response and for example to the change of therapy. For the imatinib-resistant point mutation in *PDGFRA *exon 18, tumors in which this mutation was missed to insufficient methodology may be treated with imatinib without any positive effect. The same holds true for treatment prediction in cases with false-positive results.

In order to check the quality of external mutation analyses which are now performed in an increasing number of laboratories, we decided to design an external trial being available via the initiative for quality assurance in pathology (QuiP) of the German Society of Pathology and the Professional Association of German Pathologists. The first results of this external trial underline the need of further activities in these areas as only 11 of the 19 participants were able to perform the trial successfully. Five of the eight institutions that failed ordered a new test set in the mean-time. Four of them succeeded in the repetition, in the remaining institution the second round of the external trial is still in progress.

Summarizing the results of the three inter-laboratory trials, the discussions at our four panel meetings and the results of our external trial, we developed a proposal for the mutation testing procedure which is available as additional files. We decided to propose not only one method but in some steps several different if more than one turned out to be suitable.

## Conclusions

In summary, mutation analysis in gastrointestinal stromal tumors gets increasing relevance for prognosis and treatment strategies. Our interlaboratory trials have shown that there is a broad range of possible pitfalls during the different technical procedures and also in the interpretation of results. According to our results it is mandatory that laboratories analysing GISTs should participate in interlaboratory tests on a regular basis to minimize possible mistakes. Furthermore, centralisation of mutation analysis in experienced laboratories with at least 50 analyzed tumors per year could help to increase the reliability of results.

## Methods

### Design of study

The internal trial was performed in three steps to identify potential pitfalls in methodology. Samples of paraffin-embedded GISTs were obtained from all six pathology archives of our panel in accordance with institutional review board regulations of all six institutions. We started with a set of extracted DNA samples from formalin-fixed paraffin-embedded (FFPE) tissue which were sent to all labs without any recommendations on how to perform analysis and how to report the results. In a second step each lab provided two paraffin blocks leading to a series of 12 blocks which were sent to each lab to perform the whole protocol from extraction to the final result. A third trial was added to compare the different primer sets for *c-KIT *exon 11. It is well accepted that the different mutational subtypes in this region are the most heterogeneous ones. Primer sets for *KIT *exon 9 and *PDGFRA *exon 18 were not tested in this step. Comparison of the different primer sets used within the panel for amplification of these two hot spots led to the recommendation of two different primer pairs each. Technical details for DNA extraction, the different PCR approaches, cycling profiles and bidirectional sequencing which are not described in the following study steps are provided in the additional files.

In detail, the following trials were performed:

#### 1. Comparison of mutational analysis on extracted DNA samples

The first trial was performed in order to compare the reproducibility of the mutation analysis and the reporting between the different panel labs (referred to as Lab A to Lab F in the following text) in a defined series of cases on extracted DNA samples. A set of ten DNA samples (T1.1-T1.10) extracted from formalin-fixed paraffin-embedded (FFPE) tissue provided by Lab A was sent to the panel laboratories B-F for the examination of the mutational status of *KIT *and *PDGFRA*. The integrity of DNA was confirmed by agarose gel electrophoresis. All samples were previously examined for mutations in the relevant exons. During the respective trial the mutational analysis was repeated also by Lab A.

The set included samples with mutations in *KIT *exon 9 and 11, and *PDGFRA *exon 18, and samples without mutations in these exons. The samples were provided in an anonymous fashion and each laboratory received enough material to analyze all exons mentioned above. Time limit for the reporting of results was two months, results were reported without given reporting rules.

#### 2. Mutation analysis from FFPE material

In the second trial, each group (Labs A-F) supplied two paraffin blocks (T2.1-T2.12) for mutational analysis in order to consider the heterogeneity of tissue blocks from different sources and to compare the divergent extraction protocols. The 12 blocks were collected in Lab A. H&E stained sections from each block were evaluated by an experienced pathologist (E.W.). In two samples a manual microdissection was needed because these samples contained both neoplastic and non-neoplastic tissue.

The whole set was sent to each panel laboratory to perform tissue sectioning, DNA extraction and mutational analysis. Mutational data should be reported according to the standard of the Human Genome Variation Society (HGVS; http://www.hgvs.org). Again the time limit was two months.

#### 3. Comparison of different primer sets for *KIT *exon 11

The third trial was restricted to the mutational analysis of *KIT *exon 11 to evaluate the quality and efficiency of different primer sets. A set of three DNA samples with different mutations in exon 11 (T3.1-T3.3) extracted from FFPE tissue was analyzed in each panel laboratory (except Lab B) using all different primer sets for exon 11 available within the panel. Again, the samples were provided by Lab A who repeated the analysis. The samples were provided in an anonymous fashion and each laboratory received enough material to perform the mutational analysis for exon 11 with all primer combinations. Each laboratory used its own amplification and sequencing conditions. Reporting of mutational data and time limit was the same as for the second trial.

### External trial

In 2009 an external trial was conducted by the six panel institutions. A pool of 30 paraffin embedded tissue blocks was collected including cases with *KIT *exon 9, *KIT *exon 11 and *PDGFRA *exon 18 mutations as well as cases without mutations in these three exons. Each panel lab provided blocks for this pool. All mutational data were cross-validated by at least two of the panel labs. The first nineteen participating external labs received one H&E stained section and three sections of five blocks to perform DNA extraction and mutation analysis of *KIT *exon 9 and 11 and *PDGFRA *exon 18. Additionally, each external lab received ten electropherograms from mutation analyses performed previously on these three exons to test the correct reporting of sequence data according to the HGVS standard. The panel provided each participant with the recommendation of a standard operating procedure.

## Competing interests

WD, LF, AK, GM, RP, RSS and RS declare that they have no competing interests. SMB has received funding from Novartis Oncology and Roche Applied Science and honoraria from Roche Pharma and Astra Zeneca. AG has received honoraries from Novartis Oncology. FH is funded for non-overlapping projects by Novartis Oncology. HUS has received reimbursements, honoraria and funding from Novartis Oncology and reimbursements and honoraria from Roche Pharma. EW is supported in research projects and has received honoraria by Novartis Oncology and PharmaMar.

## Authors' contributions

SMB, WD, FH, JK, AK, RP, RS, and RSS carried out the molecular genetic studies and participated in the sequence alignment. LF, AG, JK, AK, GM, HUS and EW participated in the sequence alignment, in the design of the study and performed the statistical analysis. EW, AG, GM, LF, FH and HUS participated in the diagnosis and selection of tumor material. SMB coordinated the study and drafted the manuscript together with EW and HUS. All authors revised the manuscript for important intellectual content and approved the final manuscript.

## Pre-publication history

The pre-publication history for this paper can be accessed here:

http://www.biomedcentral.com/1471-2350/11/106/prepub

## Supplementary Material

Additional file 1 **Methods used for mutation analysis by the panel labs**. This file describes how to extract DNA from formalin-fixed paraffin-embedded tissue, how PCR amplification and purification of PCR products prior to cycle sequencing can be performed and how cycle sequencing and precipitation of sequencing products should be done. Click here for file

Additional file 2 **Proposed standard operation procedure for testing and reporting of common KIT and PDGFRA mutations in GIST**. The fundamental rules that should be the gold standard in every diagnostic molecular pathology laboratory are explained. Furthermore, advices are given for the evaluation of the tissue area to be analyzed and about the procedure of sectioning and microdissecting of formalin-fixed, paraffin-embedded tissue blocks. Protocols are given for DNA extraction and quantification, for primers for KIT exons 9 and 11 and for PDGFRA exon 18 and for PCR amplification. Another protocol explains how to purify the PCR fragments and how to perform cycle sequencing. Click here for file

Additional file 3 **Description of sequence data**. This file explains how to report the sequence data. Several examples are given. Click here for file

Additional file 4 **Tables AF1-4**. The four tables contain technical details about DNA extraction, primer sets, conditions for PCR ampiflications, purification of PDR products and conditions for cycle sequencing. Table AF 1. Kits used for DNA extraction. Table AF 2. Primersets and conditions for PCR amplification of *KIT *exon 9 and 11 and *PDGFRA *exon 18. Table AF 3. Purification of PCR products prior to cycle sequencing. Table AF 4. Kits, devices and conditions used for cycle sequencing. Click here for file
