# Supplementary material for: Pitfalls in mutational testing and reporting of common KIT and PDGFRA mutations in gastrointestinal stromal tumors
Source: BMC Med Genet. 2010 Jul 4;11:106. doi: 10.1186/1471-2350-11-106 (PMC2910708; doi:10.1186/1471-2350-11-106)
Supplement: Additional file 1 — Methods used for mutation analysis by the panel labs. This file describes how to extract DNA from formalin-fixed paraffin-embedded tissue, how PCR amplification and purification of PCR products prior to cycle sequencing can be performed and how cycle sequencing and precipitation of sequencing products should be done. [file 1471-2350-11-106-S1.RTF]

AF 1. Methods used for mutation analysis by the panel labs

AF 1.1. Extraction of DNA from formalin-fixed paraffin-embedded tissue
Two 10 m thick sections were cut from each tissue block and either collected in 1.5 ml reaction tubes or mounted on slides. Paraffin was removed by xylene treatment followed by washing the slides with ethanol. Depending on the lab, this procedure was repeated up to three times. After drying tissue lysis was performed by proteinase K digestion. The kits used for further DNA extraction and purification differed between the panel labs and are listed in Table AF 1. All kits were used according to the manufacturers' instructions.
The amount and integrity of extracted DNA were estimated semi-quantitatively by agarose-gelelectrophoresis.


AF 1.2. PCR amplification and purification of PCR products prior to cycle sequencing
In order to obtain enough material for cycle sequencing, the relevant exons were amplified by PCR. The panel labs used different primer-sets which are specified for KIT exon 9 and exon 11 and PDGFRA exon 18 in  Table AF 2. Concerning PCR approaches and cycling profiles the PCR protocols of all panel labs followed standard conditions. Parameters as annealing temperatures, MgCl2 concentration and the polymerases used for PCR amplification differed and are also detailed in Table AF 2. To rule out contaminations a negative control with distilled water instead of DNA was included in each PCR run. Prior to cycle sequencing unbound primers were removed using different methods or kits (Table AF 3). 


AF 1.3. Cycle sequencing and precipitation of sequencing products
In all panel labs except Lab B employing an external core facility, bidirectional sequencing was carried out in-house using the dideoxy chain termination method of Sanger. The different fluorescence labelled dye-terminators and the devices used for electrophoresis are listed Table AF 4. For the bidirectional cycle sequencing reactions the same primers as for the primary PCR amplification were used in all laboratories except Lab B. The annealing temperatures and cycle numbers differed from the primary PCR protocol and are also specified in Table AF 4. In all panel labs except Lab E who applies CentriSep Spin Columns (empBiotech, Berlin, Germany), the cycling products were precipitated with standard sodium-acetate/ethanol precipitation. All kits were used according to the manufacturers' instructions.
