# Supplementary material for: Pitfalls in mutational testing and reporting of common KIT and PDGFRA mutations in gastrointestinal stromal tumors
Source: BMC Med Genet. 2010 Jul 4;11:106. doi: 10.1186/1471-2350-11-106 (PMC2910708; doi:10.1186/1471-2350-11-106)
Supplement: Additional file 2 — Proposed standard operation procedure for testing and reporting of common KIT and PDGFRA mutations in GIST. The fundamental rules that should be the gold standard in every diagnostic molecular pathology laboratory are explained. Furthermore, advices are given for the evaluation of the tissue area to be analyzed and about the procedure of sectioning and microdissecting of formalin-fixed, paraffin-embedded tissue blocks. Protocols are given for DNA extraction and quantification, for primers for KIT exons 9 and 11 and for PDGFRA exon 18 and for PCR amplification. Another protocol explains how to purify the PCR fragments and how to perform cycle sequencing. [file 1471-2350-11-106-S2.DOC]

**AF 2. Proposed standard operation procedure for testing and reporting of common KIT and PDGFRA mutations in GIST**

In general, as PCR amplification is a highly sensitive method and susceptible to carry over contaminations, the following fundamental rules should be the gold standard in every diagnostic molecular pathology laboratory. The working area should be divided in pre- and post-PCR sections, optimally three independent rooms for extracting DNA, preparing the PCR and performing the post-PCR analysis. Each section should have assigned equipments and reagents. Plugged pipette tips have to be used throughout and gloves to be changed between the working sections.

## AF 2.1. Evaluation of the tissue area to be analyzed

An experienced pathologist has to evaluate H&E and immunohistochemical staining and to define the most appropriate tissue block to be tested. Furthermore he has to mark on the H&E stained section the tumor area for DNA extraction to allow manual microdissection for reducing the amount of non-tumorous tissue.

## AF 2.2. Sectioning of formalin-fixed, paraffin-embedded tissue blocks

For cutting the paraffin blocks by microtome, some rules have to be observed to avoid contamination between blocks:

- blocks that are proposed for the same analysis should not be cut consecutively

- the microtome and the working area should be cleaned from paraffin material after each block

- depending on the type of microtome, the knives should be removed or relocated after each block

- if the sections are mounted on slides for manual microdissection, the water-bath should be cleaned regularly with filter-paper and the water should be changed as often as possible

Two different methods can be used to perform manual microdissection: The marking of the H&E stained slide is transferred to the tissue block before cutting one to six 10 μm thick slides in a reaction tube. This method impairs the quality of tissue blocks for further investigations. The alternative protocol is to prepare tissue sections first and to perform the manual microdissection on glass slides after deparaffinization. The slides have to be incubated for at least 30 min at 60°C before deparaffinization.

The number of slides needed for DNA extraction varies depending on the tissue area marked by the pathologist.

## AF 2.3. DNA extraction and quantification

Depending on the method chosen for manual microdissection two different protocols for deparaffinization are possible:

## AF 2.3.1. Deparaffinization of tissue sections prior to macrodissection

- incubate slides twice for 10 minutes in xylol and ethanol, respectively, followed by rehydration in 100%, 96%, 80%, and 70% ethanol for 5 minutes each

- scrape areas marked on the H&E stained section from the deparaffinized slides and transfer into a reaction tube

- centrifuge for 5 minutes at 13000 rpm; discard supernatant

- resuspend pellet in the appropriate buffer and continue with purification protocol

## AF 2.3.2. Deparaffinization of tissue sections cut into an reaction tube

- add 1 ml of xylol to the reaction tube, vortex and incubate at room temperature for 10 minutes

- centrifuge for 5 minutes at 13000 rpm; discard supernatant

- repeat the first two steps

- resuspend pellet in 1 ml ethanol, vortex and incubate at room temperature for 10 minutes

- centrifuge for 5 minutes at 13000 rpm; discard supernatant

- repeat the last two steps

- dry pellet in the open reaction tube for 10 minutes at 37°C, resuspend pellet in suitable buffer and continue with purification protocol

The deparaffinization is followed by tissue lysis with the appropriate buffers and proteinase K. After proteolysis, DNA purification is indispensable. A number of DNA purification methods has been described to date mostly being offered commercially by several companies. The kits which can be recommended due to the own experience of the panel labs are listed in Table AF 1.

For the assessment of DNA amount and the extent of degradation we highly recommend to use agarose gelelectrophoresis. Because the PCR result depends on the number of amplifiable fragments it should be avoided to use standard DNA amounts as PCR template. It is strongly recommended not to analyze samples with bad DNA quality. In such cases, additional material (e.g. fresh frozen tissue, if available, or another paraffin block) should be used.

## AF 2.4. PCR for amplification of KIT exon 9 and 11 and PDGFRA exon 18

Each laboratory offering the mutational analysis of GIST should at least perform sequencing of *KIT* exon 9 and 11 and *PDGFRA* exon 18. From the results of our trials, we decided to recommend two different primer sets for each exon:

*KIT* exon 9:

A-9 forward agtgcattcaagcacaatgg

A-9 reverse gacagagcctaaacatcccc

B-9 forward cagggcttttgttttcttcc

B-9 reverse atcatgactgatatggtagacagagc

*KIT* exon11:

A/B-11 forward gtgctctaatgactgagac

A/B-11 reverse tacccaaaaaggtgacatgg

C/D-11 forward ccagagtgctctaatgactg

C/D-11 reverse agcccctgtttcatactgac

*PDGFRA* exon 18:

A-18 forward catggatcagccagtcttgc

A-18 reverse tgaaggaggagtagcctgac

B-18 forward cagctacagatggcttgatc

B-18 reverse gaaggaggatgagcctgac

A standard PCR protocol for amplification with all recommended primer pairs is as follows:

*PCR set-up*

template: 1-20 µl (according to gelelectrophoresis)

nucleotide: 100 μM

reaction buffer: 5 μl

forward primer: 0,4 μM

reverse primer: 0,4 μM

polymerase enzyme: 1 U

distilled water: up to 50 µl

## PCR program

first denaturation 94°C 3 min

denaturation 94°C 40 sec

annealing s. o. 40 sec 40 cycles

extension 72°C 40 sec

final extension 72°C 5 min

For each amplification experiment, positive and negative controls should be carried along. A sample with water instead of DNA serves as negative control; a positive control may be DNA extracted from FFPE tissue which was amplified successfully in a previous approach. The control reactions should be checked by agarose gelelectrophoresis. The number of PCR cycles should not exceed 40 cycles.

If amplification failed twice, the analysis should be stopped at this point. The analysis can be re-tried with another paraffin block.

## AF 2.5. Purification of PCR fragments

Unbound primers and an excess of nucleotides have to be separated from the PCR fragments prior to cycle sequencing. This can be either done by polyethylenglycol (PEG) precipitation or with commercially available DNA purification columns. An overview over the methods used within the panel is given in Table AF 3. The protocol for the PEG purification is as follows:

*AF 2.5.1. Purification of PCR fragments by polyethylen glycol (PEG) precipitation*

- mix equal volumina of PCR product and PEG mix

- vortex and incubate for 10 minutes at room temperature

- centrifuge for 10 minutes at 13.000 rpm

- discard supernatant

- add 100 μl 100% ethanol, DON’T VORTEX

- centrifuge for 10 minutes at 13.000 rpm

- discard supernatant

- dry pellet at room temperature

- resuspend pellet in distilled water

- PEG-mix: 52,4 g PEG 8000

40 ml 3M NaOAc pH5,2

1,32 ml 1M MgCl_2_

add distilled water up to 200 ml

## AF 2.5.2. Cycle sequencing and precipitation

The cycle sequencing should always be performed as bidirectional sequencing and may be done with different sequencing kits. Some examples are listed in Table AF 4. The cycle sequencing products may be either precipitated or narrowed using spin columns.

Exemplarily, the cycle sequencing protocol for working with the BigDye Terminator Cycle Sequencing Ready Reaction Kit (Applied Biosystems, Darmstadt, Germany) as well as the protocol for precipitation of cycling products is given:

*AF 2.5.3. Standard PCR protocol for cycle sequencing*

Cycle sequencing set-up

template: 1-8 µl (according to gelelectrophoresis)

primer (forward OR reverse): 10 pmol

terminator ready reaction mix: 1 µl

buffer 2 μl

distilled water: ad 20 µl

PCR program for cycle-sequencing

first denaturation 1 min 96°C

denaturation 10 sec 96°C

annealing 5 sec 55/60°C* 25 cycles

extension 4 min 60°C

cooling down until precipitation 4°C

**KIT* exon 9 and 11: 55°C; *PDGFRA* exon 18: 60°C

*AF 2.5.4. Protocol for precipitation of cycle sequencing products*

- mix the following components in a 1,5 ml reaction tube and vortex

80 µl distilled water HPLC grade

10 µl 3 M sodium acetate, pH4,6

250 µl 100% ethanol

product of sequencing reaction

- centrifuge at room temperature for 15 minutes at 13.000 rpm, discard supernatant

- add 250 μl ethanol, vortex

- centrifuge at room temperature for 5 minutes at 13.000 rpm, discard supernatant

- dry pellet for 30 minutes

- freeze at -20°C or use directly for electrophoresis

If necessary, the purified cycling products are diluted prior to electrophoresis. Most conveniently, the separation of products is done by capillary electrophoresis, but depending on the available laboratory equipment it is also possible to perform polyacrylamide gelelectrophoresis. Generally these methods should be performed according to the manufacturers’ instructions.
