# Supplementary material for: Pitfalls in mutational testing and reporting of common KIT and PDGFRA mutations in gastrointestinal stromal tumors
Source: BMC Med Genet. 2010 Jul 4;11:106. doi: 10.1186/1471-2350-11-106 (PMC2910708; doi:10.1186/1471-2350-11-106)
Supplement: Additional file 3 — Description of sequence data. This file explains how to report the sequence data. Several examples are given. [file 1471-2350-11-106-S3.DOC]

### **AF 3. Description of sequence data**

For diagnostic mutation analysis DNA changes should be related to the cDNA sequence. The first nucleotide refers to the “A” from the ATG initiator methionine codon. The DNA sequence change is preceded by “c.” standing for coding cDNA sequence. Substitutions at DNA level are designated by “>” (e.g.: c.1669T>A denotes a missense substitution at nucleotide 1669 in the coding DNA sequence where a Thymidine was changed to an Adenine). Deletions are designated by “del” after the deleted amino acid or a deleted region followed by the deleted nucleotides (e.g.: c.1669_1683del15 denotes a deletion of the 15 nucleotides 1669 – 1683 in the coding DNA sequence). Accordingly, insertions are designated by “ins”.

Amino acid changes are described using the single letter amino acid code. The amino acid is preceded by “p.”. For example, a substitution of Valin 559 by Aspartic acid is designated as “p.V559D”. Deletions are designated by “del”, the “_” symbol should be used to separate the first from the last affected amino acid (e.g.: “p.W557_E561del” denotes a deletion of the five amino acids 557 – 561).
