# Supplementary material for: Pitfalls in mutational testing and reporting of common KIT and PDGFRA mutations in gastrointestinal stromal tumors
Source: BMC Med Genet. 2010 Jul 4;11:106. doi: 10.1186/1471-2350-11-106 (PMC2910708; doi:10.1186/1471-2350-11-106)
Supplement: Additional file 4 — Tables AF1-4. The four tables contain technical details about DNA extraction, primer sets, conditions for PCR ampiflications, purification of PDR products and conditions for cycle sequencing. Table AF 1. Kits used for DNA extraction. Table AF 2. Primersets and conditions for PCR amplification of KIT exon 9 and 11 and PDGFRA exon 18. Table AF 3. Purification of PCR products prior to cycle sequencing. Table AF 4. Kits, devices and conditions used for cycle sequencing. [file 1471-2350-11-106-S4.DOC]

**Additional file 4. Tables AF1-4.**

**Table AF 1. Kits used for DNA extraction**

| Lab | Kit for DNA extraction |
| --- | --- |
| A | BioRobot M48/ QIAmp DNA Mini-Kit (Qiagen, Hilden, Germany) |
| B | QIAmp DNA Mini-Kit (Qiagen, Hilden, Germany) |
| C | QIAmp DNA Mini-Kit (Qiagen, Hilden, Germany) |
| D | Pure Gene Tissue Kit (Qiagen, Hilden, Germany) |
| E | Nucleospin Tissue Kit (Macherey & Nagel, Düren, Germany) |
| F | Magna Pure (Roche, Mannheim, Germany) |

**Table AF 2. Primersets and conditions for PCR amplification of *KIT* exon 9 and 11 and *PDGFRA*** exon18

| Lab | Exon | forward primer (5’-3’) | PCR enzyme | annealing  temp. [°C] | no. of cycle | length  in bp |
| --- | --- | --- | --- | --- | --- | --- |
|  |  | reverse primer (5’-3) |  |  |  |  |
| A | 9 | agtgcattcaagcacaatgg | HotStar Taq (Qiagen, Hilden, Germany) | 57 | 40 | 146 |
|  |  | gacagagcctaaacatcccc |  |  |  |  |
|  | 11 | gtgctctaatgactgagac |  | 57 | 40 | 232 |
|  |  | tacccaaaaaggtgacatgg |  |  |  |  |
|  | 18 | catggatcagccagtcttgc |  | 60 | 40 | 256 |
|  |  | tgaaggaggagtagcctgac |  |  |  |  |
| B | 9 | cagggcttttgttttcttcc | Platinum Taq  (Invitrogen, Karlsruhe, Germany) | 57 | 45 | 266 |
|  |  | atcatgactgatatggtagacagagc |  |  |  |  |
|  | 11 | gtgctctaatgactgagac |  | 57 | 45 | 232 |
|  |  | tacccaaaaaggtgacatgg |  |  |  |  |
|  | 18 | cagctacagatggcttgatc |  | 57 | 40 | 213 |
|  |  | gaaggaggatgagcctgac |  |  |  |  |
| C | 9 | tcctagagtagcagggctt | AmpliTaq Gold  (Applied Biosystems, Darmstadt, Germany) | 54 | 50 | 284 |
|  |  | tggtagacagagctaacatcc |  |  |  |  |
|  | 11 | ccagagtgctctaatgactg |  | 54 | 50 | 215 |
|  |  | agcccctgtttcatactgac |  |  |  |  |
|  | 18 | cagggtgatgctattcagc |  | 54 | 50 | 238 |
|  |  | gattaaagtgaagaggatgagc |  |  |  |  |
| D | 9 | gccacatcccaagtgttttatg | Fideliti Taq (USB, Cleveland, USA) | 55 | 40 | 310 |
|  |  | gagcctaaacatccccttaaattg |  |  |  |  |
|  | 11 | ccagagtgctctaatgactg |  | 60 | 40 | 215 |
|  |  | agcccctgtttcatactgac |  |  |  |  |
|  | 18 | tcttgcaggggtgatgctat |  | 55 | 40 | 265 |
|  |  | agaagcaacacctgactttagagatta |  |  |  |  |
| E | 9 | cta gag taa gcc agg gct ttt gtt | Hot Goldstar (Eurogentec, Köln, Germany) | 64 | 35 | 270 |
|  |  | cct aaa cat ccc ctt aaa ttg gat t |  |  |  |  |
|  | 11 | aaaggtgatctatttttccctttctc |  | 64 | 35 | 199 |
|  |  | ccaaaaaggtgacatggaaagc |  |  |  |  |
|  | 18 | cag ggg tga tgc tat atc agc |  | 64 | 35 | 214 |
|  |  | gtc cag tgt ggg aag tgt gga c |  |  |  |  |
| F | 9 | tcctagagtaagccagggctt | Fermentas Taq (Fermentas, St.Leon-Roth, Germany) | 65-55  55 | 10  30 | 294 |
|  |  | tggtagacagagcctaaacatcc |  |  |  |  |
|  | 11 | gatgattctgacctacaaat |  | 65-55  55 | 10  30 | 342 |
|  |  | aggaagccactggagttcctt |  |  |  |  |
|  | 18 | accatggatcagccagtctt |  | 65-55  55 | 10  30 | 264 |
|  |  | tgaaggaggatgagcctgacc |  |  |  |  |

Table AF 3. Purification of PCR products prior to cycle sequencing

| Lab | Purification Method | Purification Kit |
| --- | --- | --- |
| A | polyethylenglycol precipitation | - |
| B | - | Wizard columns (Promega, Madison, USA) |
| C | - | Exo Sap-IT (USB, Cleveland, USA) |
| D | - | High Pure PCR purification kit (Roche, Mannheim, Germany) |
| E | - | Mini Elute PC purification kit (Qiagen, Hilden, Germany) |
| F | - | QIAquick PCR purification kit (Qiagen, Hilden, Germany) |

Table AF 4. Kits, devices and conditions used for cycle sequencing

| Lab | Kit | Capillary/Gel Electrophoresis | Primer | Cycling program | |
| --- | --- | --- | --- | --- | --- |
|  |  |  |  | Annealing | Cycles |
| A | Big Dye Terminator 1.1 (Applied Biosystems) | ABI 3130 Genetic Analyzer (Applied Biosystems) | as above | Exon 9: 55°C  Exon 11: 55°C  Exon 18: 60°C | 25  25  25 |
| B | Sequencing is done in an external lab (SeqLab, Göttingen, Germany) | | | | |
| C | Big Dye Terminator 1.1 (Applied Biosystems) | ABI 3130 Genetic Analyzer (Applied Biosystems) | as above | Exon 9: 54°C  Exon 11: 54°C  Exon 18:58°C | 25  25  25 |
| D | DYEnamic ET Terminator Cycle Sequencing Kit (GE Healthcare) | ABI 377 DNA Sequencer (Applied Biosystems) | as above | Exon 9: 50°C  Exon 11: 50°C  Exon 18: 50°C | 25  25  25 |
| E | Big Dye Terminator 1.1 (Applied Biosystems) | ABI 3100 Genetic Analyzer (Applied Biosystems) | as above | Exon 9: 50°C  Exon 11: 50°C  Exon 18: 55°C | 25  25  25 |
| F | Big Dye Terminator 1.1 (Applied Biosystems) | ABI 310 Genetic Analyzer (Applied Biosystems) | as above | Exon 9: 52°C  Exon 11: 52°C  Exon 18:52 °C | 25  25  25 |
